# Is there a safe no radiation option for endoscopic kidney stone treatment in children? multicenter results of modified retrograde intrarenal surgery without fluoroscopy in pediatric patients

**DOI:** 10.1007/s00240-025-01719-y

**Published:** 2025-03-05

**Authors:** Tunc Ozan, Ahmet Karakeci, Kemal Yilmaz, Necip Pirincci, Fatih Osmanlioglu, Ercan Yuvanc, Erdal Yilmaz, Irfan Orhan

**Affiliations:** 1 Firat University Medical Faculty, Department for Urology, Elazig, Turkey; 2 Kirikkale University Medical Faculty, Department for Urology, Kirikkale, Turkey

**Keywords:** Pediatric urolithiasis, Retrograde intrarenal surgery, Fluoroscopy

## Abstract

The practice of fluoroscopy during pediatric endoscopic kidney stone procedures requires attention because of radiation concerns that demand new treatment methods. This study aimed to present the multicentric results of single guide wire flexible ureterorenoscopy (URS) and retrograde intrarenal surgery (RIRS) procedures without fluoroscopy and an ureteral access sheath (UAS) in treating kidney stones in pediatric patients. Moreover, we aim to evaluate the efficacy and safety of this procedure to ascertain the feasibility of this radiation-free therapeutic intervention for treating kidney stones in children. A retrospective analysis was done on the data of 105 pediatric patients who underwent retrograde intrarenal surgery (RIRS) treatment in two tertiary healthcare centers without fluoroscopy and UAS between May 2014 and May 2024. Of these 105 patients evaluated, 74 (70.5%) were male and 31 (29.5%) were female. The patients had a mean age of 71 ± 4 (ranging from 6 to 204) months. The mean size of stones was 9.3 ± 5 (ranging from 6 to 20) mm, and the average operation time was 51 (ranging from 31 to 98) minutes. Additionally, in 24 (22.8%) patients, the flexible URS could not proceed through the ureteral orifice. Thus, a double J stent was inserted, and the surgical procedure was repeated one month later without any complications. However, 2 (1.9%) of the patients experienced postoperative fever, and 6 (5.7%) patients had minor complications related to haematuria. Stone-free status was observed in 87 out of 105 patients (82.9%). Despite using a single guide wire without fluoroscopy and UAS in treating kidney stones in pediatric patients, the RIRS procedure is technically effective and safe. It may be considered a viable non-surgical procedure that is effective in safeguarding pediatric patients from the harmful effects of radiation, rendering it a promising alternative for pediatric urolithiasis management.

## Introduction

The incidence of urinary stone disease is increasing worldwide, with a particularly significant increase in the pediatric population [1, 2]. An epidemiologic study on pediatric patients reported the incidence rate of asymptomatic kidney stones as 1% [3]. Kidney stone disease develops in children mostly because of urinary system failure due to anatomical anomalies, metabolic disorders, and/or recurrent urinary tract infections [4]. Hence, patients of this age group are at high risk of kidney stone relapse, necessitating exposure to repeated interventions. Kidney stones in pediatric patients are mainly treated using percutaneous nephrolithotomy (PCNL) (mini/micro) and extracorporeal shock wave lithotripsy (ESWL) [5, 6]. Even though the guidelines recommend ESWL as the first-line treatment option, alternative treatment techniques are considered because of ESWL’s disadvantages, like limited success rates, the need for multiple sessions, and lack of knowledge regarding long-term results [7, 8].

With the development of laser technology and the miniaturization of thin, flexible ureterorenoscopes, retrograde intrarenal surgery (RIRS) has become a viable option for treating renal stones in pediatric patients. Although there was hesitance in using endoscopic devices in the beginning due to the apprehensions regarding the safety and practicality of using endoscopic devices in pediatric patients, currently, since the size of the flexible ureterorenoscopy (URS) device has decreased, RIRS is being administered to kidney stone patients of pediatric age particularly those with resistance to ESWL treatment or difficulties in passing stone fragments [9].

Conventional RIRS techniques include fluoroscopy and placement of an ureteral access sheath (UAS), although the most critical step may cause complications. Recent studies suggest that the risks due to radiation exposure in pediatric patients are 2–3 times higher than in adults [10].

This study aimed to assess the efficacy and safety of the modified RIRS technique without using an UAS and fluoroscopy in order to protect surgeons and patients from radiation.

## Materials and methods

Necessary permissions to conduct this study were obtained from the local ethics committee. (2019/05/01). A retrospective analysis was conducted on data extracted from a total of 105 patients who were under the age of 18 and had undergone treatment for kidney stones with the RIRS procedure in two tertiary healthcare centers between May 2014 and May 2024 without using an UAS and fluoroscopy. All pediatric kidney stone patients of these two clinics were administered the no-UAS and no-fluoroscopy method since the onset of the RIRS technique. The diagnosis of urolithiasis was based on methods such as ultrasonography (USG) and plain Kidney-Ureter-Bladder (KUB) radiography. USG imaging technique included B and S-mode (harmonic and not harmonic) for stone detection and distinction from other surrounding tissues and color Doppler mode for inducing a twinkling effect to measure acoustic shadow width in smaller stones. In 11 cases, non-contrast computer tomography (CT) was applied wherever determination of the stone’s size and/or location was not possible with the previously mentioned imaging methods due to patient-related reasons. Stone size was calculated according to the methods recommended by the European Association of Urology [11]. The indication for RIRS was restricted to patients with a stone size of ≤ 20 mm, as ESWL treatment units in both the centers were serving only adult patients and lacked an anesthesiology team in the units, which is essential for the ESWL administration in pediatric patients. Patients with calyceal diverticulum stones, ectopic pelvic fusion abnormalities, and all pathologies with the potential to cause patient positioning difficulties during endoscopic surgery, such as cerebral palsy, genetic disorders like osteogenesis imperfekta, achondroplasia, and Marfan syndrome, or patients with bifid pelvis were excluded from the study. Patient data pertaining to operation duration, stone-free rates, sizes, and location of stones were recorded systematically. All patients included in the study submitted duly filled informed consent forms. Preoperative laboratory evaluations like serum creatinine levels, urine cultures, urinalysis, bleeding and clotting times, and the complete blood counts of the patients were documented. Negative urine cultures were obtained from all patients before surgery, and patients with positive urine cultures were surgically treated after appropriate antimicrobial medical treatment before initiating surgical intervention. All patients underwent KUB radiography on the first day and USG imaging one month after the operation. Results were classified as residual stones, clinically insignificant residual fragments (CIRF), or stone-free. The absence of any stone fragments categorized patients as stone-free. Asymptomatic, non-obstructing, non-infectious stone fragments with ≤ 4 mm size were defined as CIRFs. Symptoms causing stones or stones larger than 4 mm were classified as residual.

### Surgical technique

**Fig. 1:**
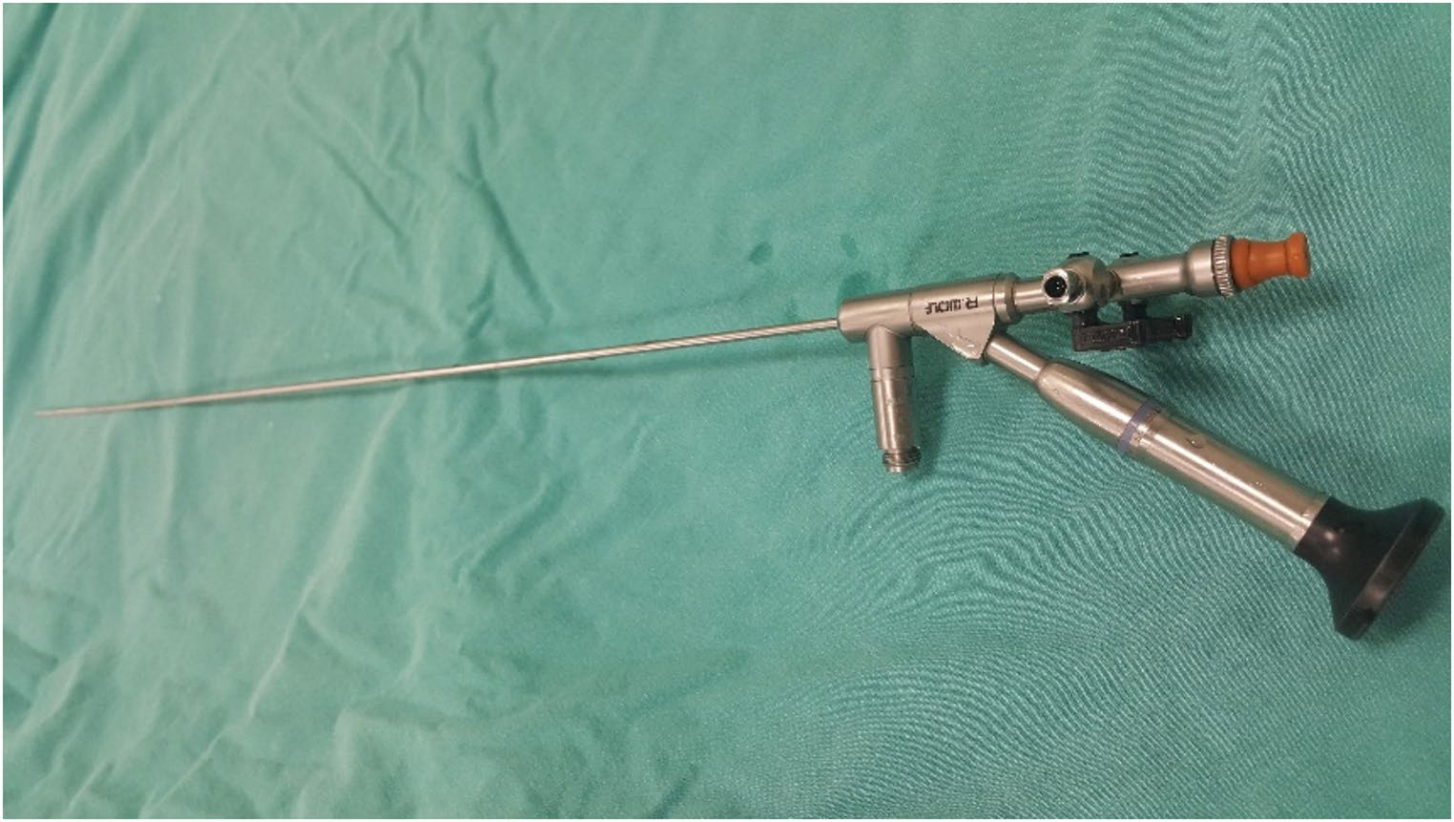
Semirigid ureterorenoscope (4.5 F, Wolf, Germany)

**Fig. 2:**
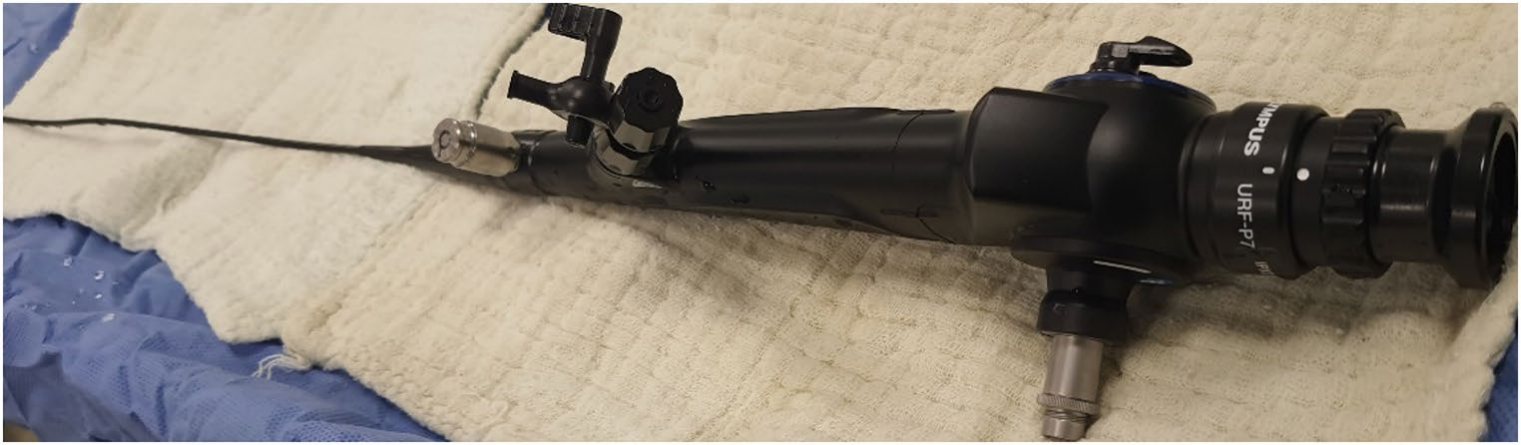
The 4.9 F flexible ureterorenoscope (Olympus Medical System URF-P7, Tokyo, Japan)

**Fig. 3:**
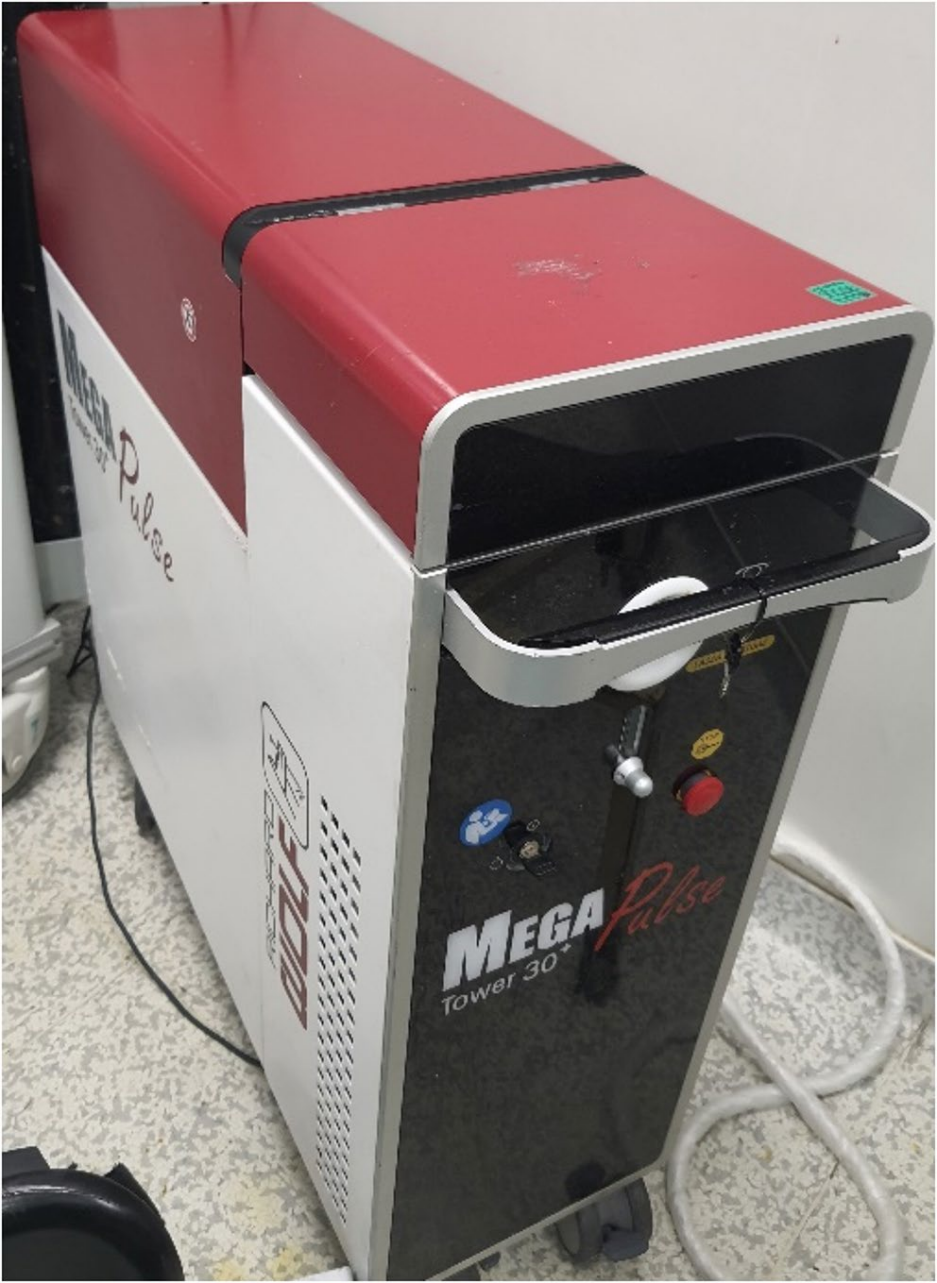
The holmium laser device (Mega Pulse 30 +, 30 watts, 4.0 joules and 25 Hz.Wolf, Germany)

The procedure was performed under general anesthesia with the patient in the lithotomy position by 5 surgeons specializing in pediatric urology and having at least 5 years of surgical experience in this field. The same type and brand label of equipment as described below were used in both centers to ensure standardization. A semirigid ureteroscope (4.5 F, Wolf, Germany) was used for the insertion of a hydrophilic guide wire into the ureter (0.038 inches, Cook Medical, Limerick, Ireland), identification of possible additional stones, and general evaluation of the ureter (Fig. 1). The tip of the hydrophilic guidewire remaining outside the patient was placed in the working channel of the flexible URS (Olympus Medical System URF-P7, Tokyo, Japan) (Fig. 2). Using the controlled tension of the guide wire, the renal pelvis was accessed under direct vision. In patients where flexible URS could not enter the ureter, ureteral balloon dilation was attempted to dilate the ureters (UroMax Ultra, Boston Scientific, USA). In cases where the URS was not feasible despite dilation, a 3 F, 20 cm double J stent (Rusch, Teleflex Medical, Westmeath, Ireland) was inserted, and the procedure was repeated after one month. The hydrophilic guidewire was removed after the flexible URS reached the renal pelvis successfully. The entire stone dusting process was done with a holmium laser device (Mega Pulse 30+, 30 watts, 4.0 joules, and 25 Hz) (Wolf, Germany) with a 272 μm laser fiber operational at a 5–10 Hz speed and an energy level of 1.0–2. 0 J (Fig. 3). Stone extraction was deliberately not performed due to the potential risks of complications during the removal process. The flexible ureterorenoscope is carefully removed with the ureter under direct observation to avoid overlooking any potential ureteral injuries at the end of the procedure. A postoperative double J stent was placed in the ureters of patients with mucosal edema, heavy stone burden (≥ 15 mm), ureteral injury, and solitary kidney. A perioperative ultrasound imaging was performed in order to confirm the location of the catheter. The modified Clavien Dindo classification was used to evaluate postoperative complications [8]. The double J placed patients underwent stent removal with a pediatric cystoscope (8/9.8 F, Wolf, Germany) after one month.

### Statistical analysis

Statistical analysis was conducted using the SPSS 22.0 program for Windows (Armonk, NY, USA). The patients’ demographic and operational data are presented as mean ± standard deviation, and median (minimum-maximum) values ​​were used in the descriptive statistics of the data. The compliance of the variables with normal data distribution was evaluated with the Kolmogorov-Smirnov test. For quantitative variables, the Kruskal-Wallis H test and then the Dunn-Bonferroni test as a post-hoc test were used in the analysis of measurements between more than two groups with significant differences, and the Mann-Whitney U test was used in the analysis of measurements between two independent groups. Spearman correlation coefficient was used to determine the correlation between two independent variables. Statistical significance was set at a p-value of less than 0.05 with a 95% confidence interval. True Editors Editing Services has completed the linguistic evaluation of the manuscript under order number 6159.

## Results

**Fig. 4:**
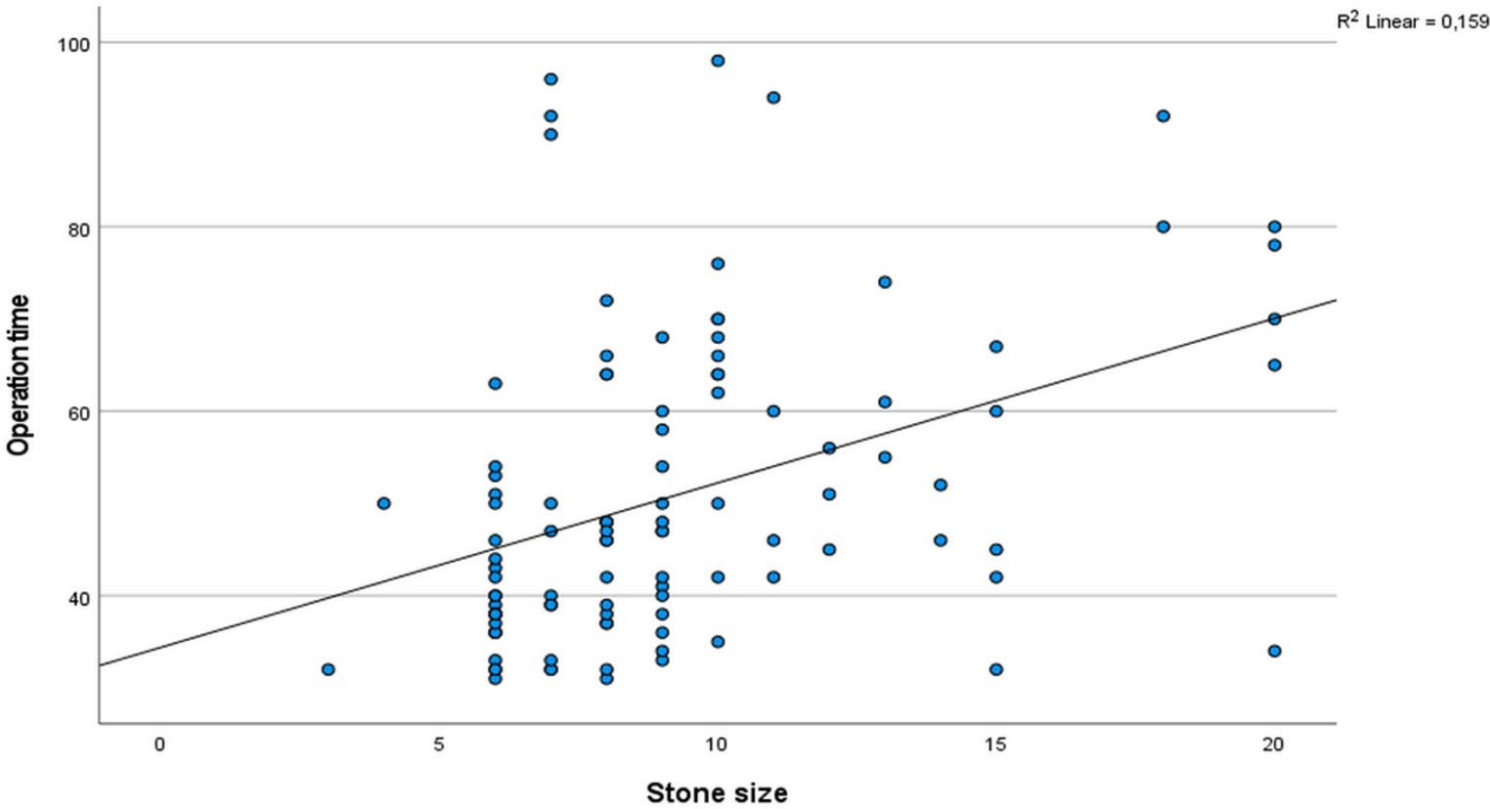
Correlation between operation time and stone size

**Fig. 5:**
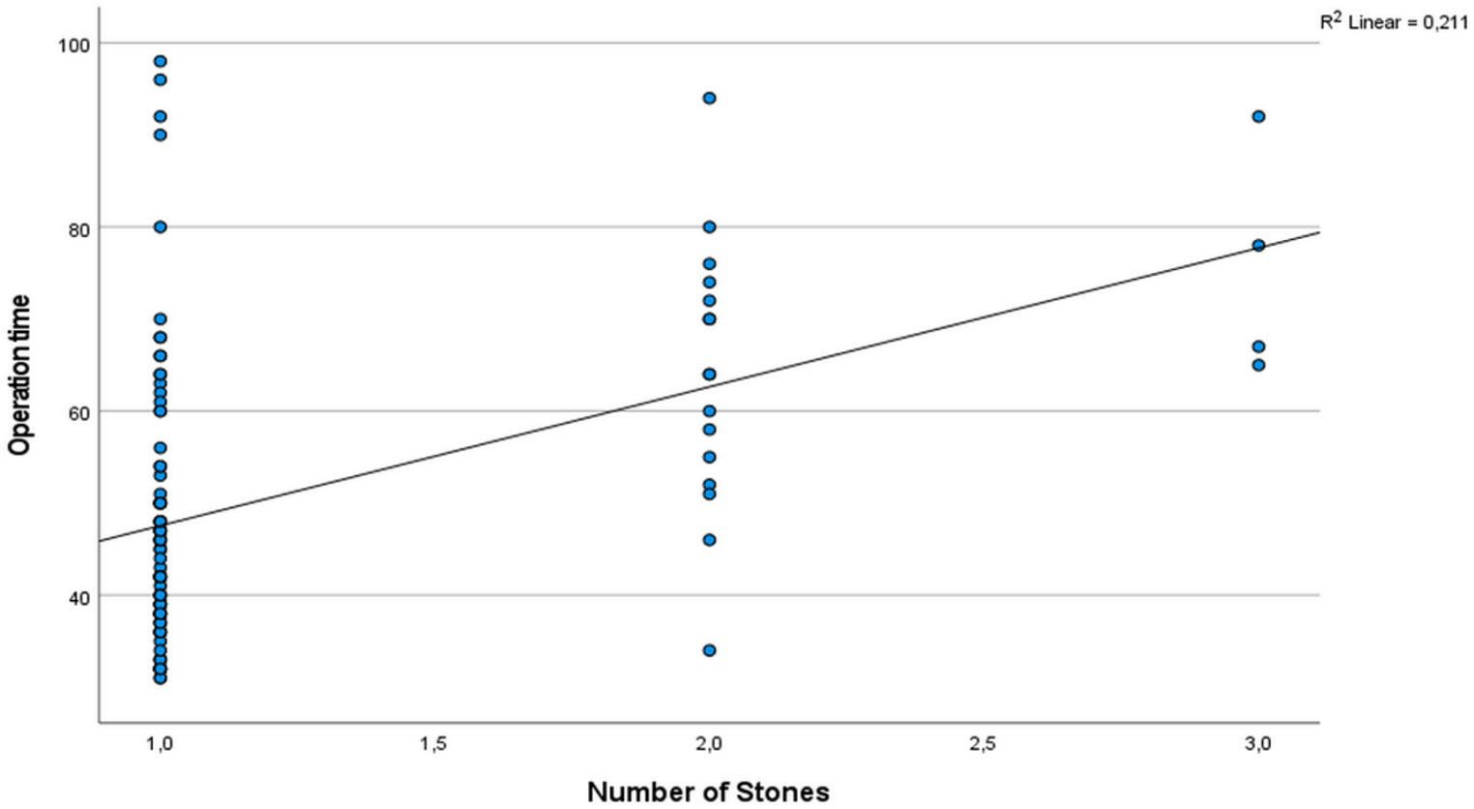
Correlation between operation time and number of stones

Of the patients, 74 (70.5%) were male and 31 (29.5%) were female. Their average age was 71 ± 4 (ranging from 6 to 204) months. The average size of the stones was 9.3 ± 5 (ranging from 6 to 20) mm, and the average duration of the operation was 51 (ranging from 31 to 98) minutes. Table 1 presents the characteristics of the stones. In 24 cases (22.8%), the flexible ureteroscopy (URS) procedure could not proceed through the ureter orifice. Therefore, a double J stent of 3 F and 20 cm was placed, and the surgery was repeated after one month. No intraoperative complications were observed in any of the patients. No complications occurred during surgery in any of the patients. Postoperative fever (Modified Clavien 1) was observed in 2 out of 105 patients (1.9%), and 6 patients (5.7%) experienced haematuria (Modified Clavien 2). Out of the total patients, 87 (82.9%) were observed to have no stones, 12 (11.4%) had small, clinically insignificant stone fragments, and 6 (5.7%) had significant stone residue. The success rates based on the location of the stone are provided in Table 2. Our study also analyzed the relationship between operation time and stone size, number, degree of hydronephrosis, and stone location. Results revealed a statistically significant relationship between the operation time, the stone size, and the number of stones (*p* < 0.05, respectively) (Figs. 4 and 5, respectively). In contrast, no statistically significant relationship was detected between operation time and the degree of hydronephrosis, the location of the stone, and preoperative double J catheter placement (p:0.610, p:0.288, p:0.585, respectively). Similarly, no statistically significant relationship was found between the operation time and the presence of a preoperative double J catheter (p:0.585). Since only 11 patients in our study had preoperative CT imaging, the relationship between stone density and operation time could not be analyzed due to the small sample size.

**Table 1:** Demographic and clinical data of the patients

Variable	No.cases (%)	Mean±SD (range)
**Age** (months)		71±4 (6-204)
**Sex**		
Male	74 (70.5)	
Female	31 (29.5)	
**Laterality**		
Left	49 (46.6)	
Right	56 (53.4)	
**Hydronephrosis**		
No Hydronephrosis	29 (27.6)	
Grade 1 Hydronephrosis	30 (28.6)	
Grade 2 Hydronephrosis	28 (26.7)	
Grade 3 Hydronephrosis	18 (17.1)	
**Stone Size** (mm)		9.3±5 (6-20)
**Stone Density** (HU)		1131±4 (343-1712)
Measured (CT applied patients)	11 (10.5)	
Not measured	94 (89.5)	
**Number of Stones**		
Single	85 (80.9)	
Two	16 (15.2)	
Three	4 (3.9)	
**Stone Location**		
Pelvis	52 (49.5)	
Lower pole	37 (35.3)	
Middle pole	9 (8.6)	
Upper pole	7 (6.6)	

*SD* Standard Deviation

**Table 2:** Success and postoperative double J placement rates according to stone location

Location	Stone- free (%)	CIRF (%)	Residual stone (%)	Total (%)	Postoperative Double J Placement
Renal pelvis	45 (51.7)	4 (36.3)	3 (42.8)	52 (49.5)	8
Lower pole	31 (35.6)	3 (27.3)	3 (42.8)	37 (35.2)	4
Middle pole	6 (6.9)	2 (18.2)	1 (14.4)	9 (8.6)	5
Upper pole	5 (5.8)	2 (18.2)	-	7 (6.7)	3
Total (%)	**87 (83)**	**11 (8.5)**	**7 (8.5)**	**105 (100)**	**20**

*CIRF* Clinically Insignificant Residual Fragments

A postoperative double J stent was placed in the ureters of 20 patients with mucosal edema (*n* = 3), heavy stone burden (*n* = 15), injury, and solitary kidney (*n* = 2). The double J stents were removed one month after the operation under general anesthesia.

## Discussion

ESWL, PNL (micro/mini), and RIRS are considered well-established, acceptable treatment options for children with kidney stones, as they have shown effective outcomes in adult patients. Pediatric patients are considered to be at high risk for recurrent kidney stone disease [1]. ESWL was first applied to adult kidney stone patients in the 1980s, and over time, it became accepted as the primary treatment for pediatric patients with kidney stones smaller than 20 mm [7]. Nevertheless, the treatment was controversial due to its detrimental effects on kidney parenchyma, with long-term results related to this effect on developing kidneys [8, 9, 12]. Due to factors such as resistance to ESWL and the need for multiple treatment sessions, there has been an increase in the exploration of alternative treatment modalities. The first data on PNL treatment in pediatric kidney stone disease management has been published by Woodside et al., incorporating similar results regarding the effectiveness of the treatment compared to that in adult patients but with a lower incidence of complications and shorter duration of hospital stay [13]. According to Desai et al.’s study [5], based on 56 pediatric PNL patients, a stone-free rate of 90% was achieved. The study also reported a correlation between the amount of bleeding and the number of renal accesses, emphasizing that the calibration of the Amplatz sheet is correlated with the amount of bleeding. The use of Amplatz sheaths smaller than 22 F was recommended in this study to minimize hemorrhagic complications. Nonetheless, injury to neighboring organs, especially complications arising while accessing renal stones in the upper calyx during the PNL procedure, has raised questions about the safety of this procedure in pediatric patients [14, 15]. The development of new optic systems, flexible URS, the intervention of the ho-YAG laser lithotripsy, and increased surgical experience have made the efficient use of RIRS feasible for treating renal stones in children. The first extensive study on the treatment of pediatric renal stones in children was published by Cannon et al. in 2007 (14). In a study involving 21 pediatric patients with lower calyx stones, a stone-free rate of 76% was reported after the RIRS procedure, with no intraoperative complications. In the same study, the authors reported that preoperative stent placement for passive dilatation was used in 38% of the cases, and a ureteral access sheath (UAS) was used during the procedure in 43% of the cases [16]. Another study, which included 100 pediatric cases, reported a stone-free rate of 91%, with ureteral perforation complications in 5 cases, necessitating ureteral reimplantation in 1 case due to ureteral stricture [17]. No intraoperative complications were reported in another study of 56 pediatric patients with renal stones less than 15 mm in diameter who underwent preoperative double J stent placement preceding the RIRS procedure. However, in the postoperative period, 3 patients suffered from urinary infection and 1 patient was exposed to macroscopic hematuria [18]. These results highlight the emerging importance of RIRS as a feasible, minimally invasive treatment option for nephrolithiasis among pediatric patients, with high stone-free rates and adequate safety.

In our series, we did not routinely insert a double J stent for passive ureteral dilation before the procedure. During the study, we refrained from using UAS due to the young mean age of our patient cohort, considering the risk of ureteral perforation. However, in 24 cases where we encountered difficulty passing through the ureteral orifice, we inserted a double J catheter for dilatation purposes and repeated the procedure one month later. Tanaka et al. reported a correlation between the success rate of the RIRS procedure, the size of the stone, and the age of the patient in a study involving 50 pediatric patients [19]. Unsal et al.’s [20] study evaluating the effectiveness of the RIRS procedure in preschool-age children (under 7 years old) reported a 100% stone-free rate for renal stones smaller than 10 mm and an 81% stone-free rate for stones larger than 10 mm. Passive ureteral dilation was required in 37.5% of incidences. This study included the youngest pediatric patient (10 months old) undergoing the RIRS procedure in the literature, with a mean stone size of 11.5 mm. In this study, UAS was used in 17.6% of cases, and one case of ureteral perforation was reported as a complication during ureteral dilatation. While routine fluoroscopy was used in all the pediatric RIRS cases mentioned above, our study executed the procedure successfully without fluoroscopic intervention. The youngest patient in our study sample was 6 months old at the time of the procedure, and to our knowledge, this was the youngest patient to undergo the RIRS procedure in the literature. Our study achieved a stone-free rate of 82.9%, which is consistent with the literature. The average operation time of 51 (31–98) minutes obtained in our study is similar to that reported in previous studies [10]. The size of the stone is the most important known risk factor affecting the duration of the operation. The average stone size in our study was 9.3 mm. Our statistical analysis has shown that the stone size and the number of stones correlate with the operation duration obtained in the study in a positive way, which was in accordance with existing literature [10, 21].

The use of fluoroscopy is essential for ensuring the safety of RIRS procedures [22]. While performing Retrograde Intrarenal Surgery (RIRS), the use of fluoroscopy is crucial for managing Ureteral Access Sheath (UAS) and stent placement, as well as addressing any residual stones and potential urinary tract perforations. However, it is important to note that fluoroscopy usage may lead to certain health risks, including infertility, genetic mutations, and increased likelihood of cancer, for both surgical teams and patients [23]. The severity of potential radiation effects is directly related to the dose and duration of exposure. Therefore, protective equipment is vital to minimize harm. Despite adopting stringent protective precautions, exposure to radiation during the RIRS procedure remains inevitable, affecting both the surgical team and patients [24]. In a study evaluating fluoroscopy time in pediatric patients undergoing RIRS, the mean fluoroscopy time was 33 ± 15 s. Although this period is relatively short compared to PNL, patients are still exposed to significant radiation. During the classic RIRS procedure, the initial step involves the placement of the guidewire catheter safely by using the semirigid URS under direct visualization until it reaches the renal pelvis. To avoid radiation exposure, we did not use fluoroscopy. The UAS placement process usually entails sliding the sheath over the guidewire catheter with the help of fluoroscopy. However, we opted for an alternative approach that did not require UAS at all. It is known that flexible URS could lead to high renal pelvic pressure (RPP) with a high probability of absorption of irrigation fluid, bacteria, and endotoxin into the bloodstream, resulting in acute complications such as systemic inflammatory response syndrome, sepsis, and long-term complication of renal function impairment. It is known that UAS could reduce the RPP to a certain extent, but it still cannot control and monitor the RPP to reduce the incidence of pressure-related complications [25]. Notably, we did not encounter any complications related to the absence of a UAS during the procedure embraced in our study. Although we did not encounter any serious complications due to increased pelvicalyceal pressure despite not using UAS in our study, there is still a need for large-scale research studies to conclude that the no-UAS method is safe and effective and is a reliable alternative to the classical fluoroscopy-guided method.

As a result, we did not need fluoroscopy, which means that there was no risk of exposure to harmful radiation. Although a similar success rate was reported in a prior study in our country where RIRS was performed without the use of fluoroscopy, our study’s superiority lies in the fact that all procedures, including guide wire placement, were performed under direct visualization. Furthermore, UAS was not included in any of the cases, and the number of cases was higher in our study [10]. The advantages of this study can be defined as being multicenter, being the largest series study conducted in pediatric patients without the use of access sheath, including the youngest age group of patients to the best of our knowledge. However, the study has limitations, including a relatively small sample size, retrospective nature, and lack of a control group. Since neither center used fluoroscopy in pediatric RIRS patients, no data regarding the method of using fluoroscopy-guided procedures in children were available, which would have been valuable for creating an age-matched control group.

## Conclusion

The RIRS procedure is a safe and technically effective way to treat kidney stones in pediatric patients. It involves using only a guide wire and an access sheet without fluoroscopy, offering a viable alternative that eliminates the harmful effects of radiation, thereby serving as a protective measure for both pediatric patients and surgeons. Despite our study results’ support for this method, further future controlled studies with larger patient cohorts are necessary to assess the long-term success and safety of the RIRS procedure without fluoroscopy in pediatric kidney stone patients.

## Data Availability

No datasets were generated or analysed during the current study.
